# Primary thyroid gland myxofibrosarcoma: a case report and review of the literature

**DOI:** 10.1186/s40792-022-01496-5

**Published:** 2022-07-25

**Authors:** Maria Chara Stylianidi, Lena Haeberle, Matthias Schott, Yuriko Mori, Christina Antke, Frederick Lars Giesel, Gerald Antoch, Irene Esposito, Wolfram Trudo Knoefel, Andreas Krieg

**Affiliations:** 1 Department of Surgery (A), Medical Faculty, Heinrich-Heine-University and University Hospital Duesseldorf, Moorenstr. 5, Bldg. 12.46, 40225 Duesseldorf, Germany; 2 Institute of Pathology, Medical Faculty, Heinrich-Heine-University and University Hospital Duesseldorf, Duesseldorf, Germany; 3 Division for Specific Endocrinology, Medical Faculty, Heinrich-Heine-University and University Hospital Duesseldorf, Duesseldorf, Germany; 4 Department of Nuclear Medicine, Medical Faculty, Heinrich-Heine-University and University Hospital Duesseldorf, Duesseldorf, Germany; 5 Department of Diagnostic and Interventional Radiology, Medical Faculty, Heinrich-Heine-University and University Hospital Duesseldorf, Duesseldorf, Germany

**Keywords:** Soft tissue sarcoma, Myxofibrosarcoma, Thyroid gland

## Abstract

**Background:**

Myxofibrosarcoma is a common soft tissue sarcoma of the extremities, which occurs very rarely in the thyroid gland.

**Case presentation:**

We report the case of a 61-year-old male who presented with a swelling of the left side of the neck and a newly emerged hoarseness. Ultrasound depicted a hypoechoic thyroid nodule with microcalcifications that was highly suspicious for malignancy. He underwent a left hemithyroidectomy. Histopathological examination and immunohistochemical studies revealed a myxofibrosarcoma of the thyroid gland.

**Conclusion:**

Myxofibrosarcoma of the thyroid gland is extremely rare. The diagnosis is based on histopathological features. Radical surgery achieving tumor-free resection margins remains the only chance for cure. However, the role of radiotherapy and/or chemotherapy is still under debate. Due to their high tendency for locoregional recurrence, a close follow-up after surgery is mandatory.

## Background

Myxofibrosarcoma (MFS) is one of the most common malignant soft-tissue neoplasms in elderly patients and has a slight male predominance [1]. MFS appear as mucoid and nodular lesions with a coarse plexiform capillary growth pattern composed of pleomorphic spindle-shaped cells in a myxoid and hypocellular background. However, in high-grade tumors more solid and cellular areas can be observed [2, 3]. Most frequently, these tumors appear within the dermis and subcutis or in the skeletal muscle of the extremities, but there are a few cases reported in which MFS is appearing in the head and neck region, including the hypopharynx [4]. Independent of the localization, complete resection of MFS with tumor-free resection margins remains the gold standard for optimal local tumor control. Whereas this therapeutic regimen might be suitable for small, low-grade or superficial tumors, for large high-grade intramuscular MFS, adjuvant chemo- or radiotherapy may be indicated. However, the 5-year local recurrence rate for MFS of the extremity is with 14.6% comparable to that of other soft tissue sarcoma (STS) subtypes [5]. To our knowledge, there are only 4 cases reported that involve the thyroid gland and that were diagnosed postoperatively (Table 1) [6–9].

**Table 1:** Synopsis of published cases of thyroid myxofibrosarcoma

Author	Patient	Symptoms	Radiological findings	FNAC	Therapy	Resection margins	Follow up
Darouassi et al. [6]	74-year-old female	Left lateral cervical swelling of 2 months evolution	Ultrasound and CT scan: tumor process in the left thyroid lobe, ipsilateral submandibular lymphadenopathy	Not mentioned	Surgery: total thyroidectomy with tumor resection and ipsilateral functional lymph-node dissection; Chemotherapy: doxorubicin and ifosfamide for recurrence (6 cycles)	Negative	Local recurrence after 1 month
Salama et al. [7]	76-year-old female	Rapidly enlarging left lower neck mass, dyspnea	Ultrasound: bilateral heterogeneous thyroid nodules, left lobe 7.1 × 4.5 cm, right lobe: 1.4 × 1.6 cm; CT-scan: left lobe: large solid mass, 7 × 6x5 cm with heterogeneous enhancement and cystic degeneration, no calcification	Spindle cell proliferation of moderate cellularity, occasional blood vessel fragments embedded in myxoid background. Moderately atypical spindle-shaped nuclei with moderate amount of ill-defined cytoplasm. Few scattered large bizarre cells with eccentric hyperchromatic nuclei and abundant cytoplasm. Cytological diagnosis: anaplastic thyroid carcinoma	Surgery: total thyroidectomy; Radiotherapy (postoperative)	Close to the circumferential margins	Not mentioned
Kouassi et al. [8]	45-year-old female	Pre-existing goiter with increasing swelling, dysphonia, hoarseness	Ultrasound: confirmation of the tumor, no lymphadenopathy	Not mentioned	Surgery: total thyroidectomy, right sternocleidomastoid muscle and laryngeal nerve resection	Not mentioned	Not mentioned
Zhang et al. [9]	65-year-old male	Not mentioned	Not mentioned	Not mentioned	Not mentioned	Not mentioned	Dead of disease

*FNAC* fine-needle aspiration cytology

## Case presentation

**Fig. 1:**
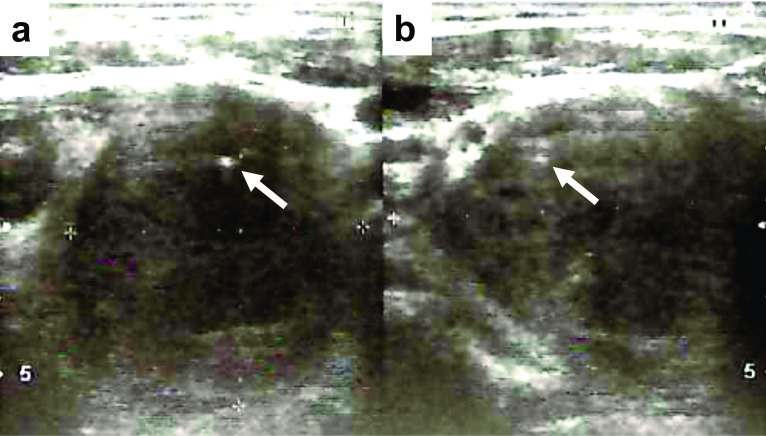
Preoperative thyroid sonography. Ultrasound revealed a large hypoechoic nodule with microcalcification (arrows) and irregular margins in the left lobe measuring 40 × 39 × 52 mm. **a** Transverse plane. **b** Sagittal plane

A 61-year-old male presented at our emergency department with a swelling of the left side of the neck that increased over a period of 4–6 weeks and hoarseness. Clinical examination revealed a clearly visible enlarged, hard, and fixed left thyroid gland. Fiberoptic laryngoscopy was able to exclude primary neoplasia of the vocal cords, but confirmed paresis of the left vocal cord. Ultrasound displayed a thyroid gland with a total volume of 60 ml (left lobe: 48 ml; right lobe: 12 ml) as well as a large hypoechoic nodule with microcalcification and irregular margins in the left lobe measuring 40 × 39 × 52 mm, which was highly suspicious for a malignant thyroid tumor (Fig. 1). Laboratory findings were as follows: TSH 1.50 µIU/ml (reference: 0.27–4.20 µIU/ml), free T4 11.9 pg/ml (reference: 9.1-0.19.1 pg/ml), free T3 3.5 ng/l (reference: 2.6–5.1 ng/l), thyroglobulin-antibody (Anti-Tg) < 20 IU/ml (reference: < 40 IU/ml), thyroid-peroxidase-antibody (Anti-TPO) 16.2 IU/ml (reference: < 35 IU/ml), TSH receptor antibody < 0.3 IU/l (reference: < 1.7 IU/l), calcitonin < 2 pg/ml (reference: < 8 pg/ml).

**Fig. 2:**
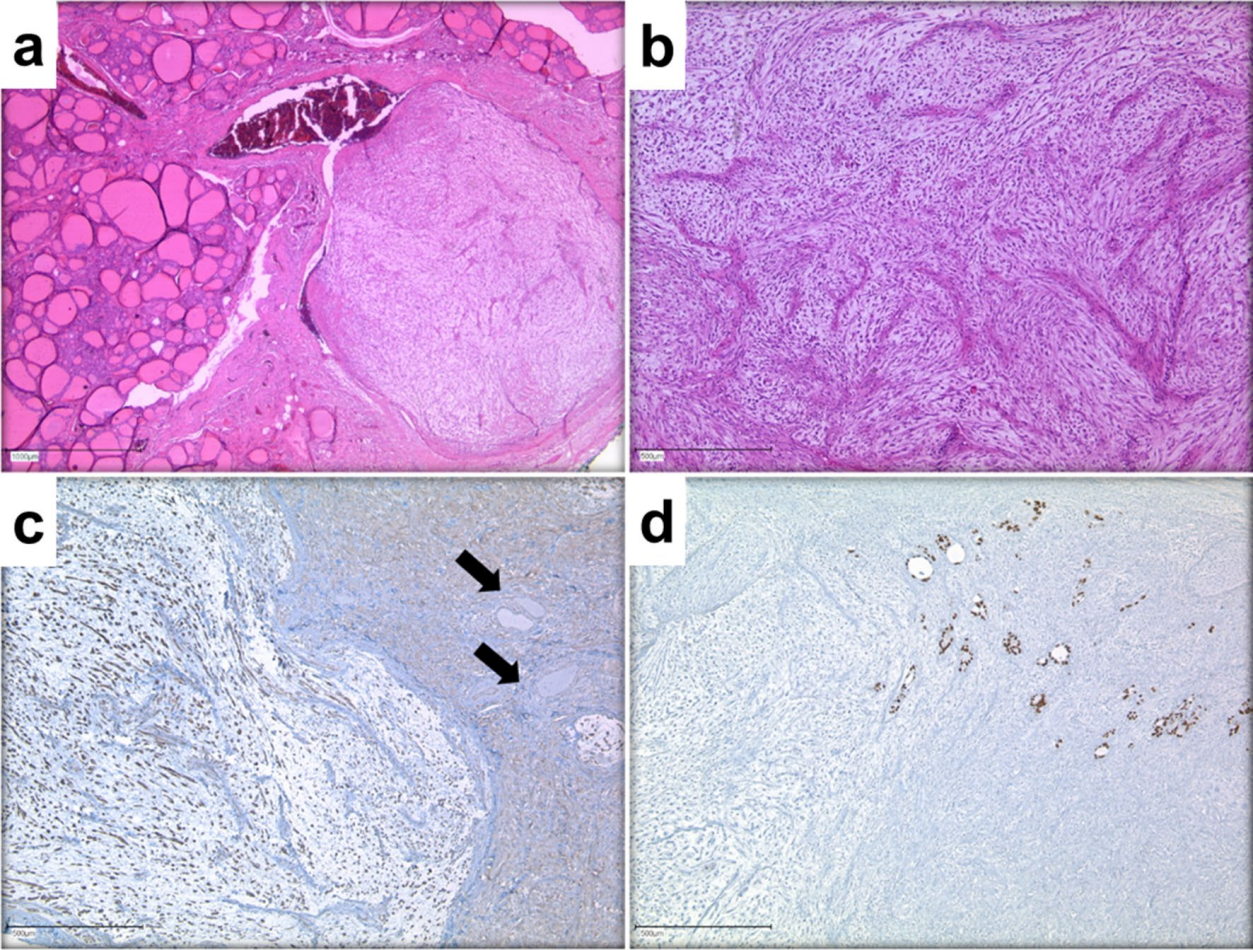
Histomorphology and immunohistochemistry of high-grade MFS of the thyroid gland. **a** MFS of the thyroid gland presenting as multi-nodular tumor composed of pleomorphic spindle-shaped cells infiltrating the normal thyroid parenchyma with vascular invasion (hematoxylin–eosin staining (HE), ×25). **b** Higher magnification demonstrates a myxoid stroma arranged along curvilinear blood vessels (HE, ×50). **c** Immunohistochemical staining showing strong expression of CD99 in myxoid (left) and more densely packed (right) tumor areas, while remnant thyroid follicles (arrows) stain negative for CD99 (×50). **d** Remnant thyroid follicles (upper right) exhibit a strong nuclear expression of TTF-1, while surrounding tumor cells remain negative (50x)

Although fine-needle aspiration with cytology is the gold standard in the evaluation of suspicious thyroid nodules, we decided against it because of the new onset hoarseness suggestive of a locally invasive process and ultrasound findings indicative of a rapidly growing thyroid carcinoma, and performed a left hemithyroidectomy with central lymphadenectomy under curative intend. Intraoperative invasion of the esophageal muscle was noted, requiring tangential resection of the muscle. Importantly, all resection margins were free of tumor on intraoperative frozen section examination. Histopathological examination revealed a partial necrotic mesenchymal tumor with capsular invasion and blood vessel infiltration that spread into the perithyroidal soft tissue. Immunohistochemical staining was positive for vimentin, SMA and CD10, and partially positive for CD68/KP1, but negative for Cdk4, MDM2, Bc12, Muc-4, S100, desmin, CD34, EMA, CK AE1/3, TTF1 and TLE-1. In addition, Ki67 labeling index was up to 80% in tumor hotspot areas and immunohistochemistry showed a strong expression of CD99 in myxoid and more densely packed tumor areas, whereas staining of the remaining thyroid follicles was negative. Thus, the combination of morphological and immunohistochemical aspects indicated a high-grade MFS (Fig. 2). Moreover, a total of 8 lymph nodes were resected, and a 0.1-cm lymph node metastasis of the tumor was detected in one lymph node. Importantly, final histopathology confirmed the complete resection of the tumor with negative resection margins.

**Fig. 3:**
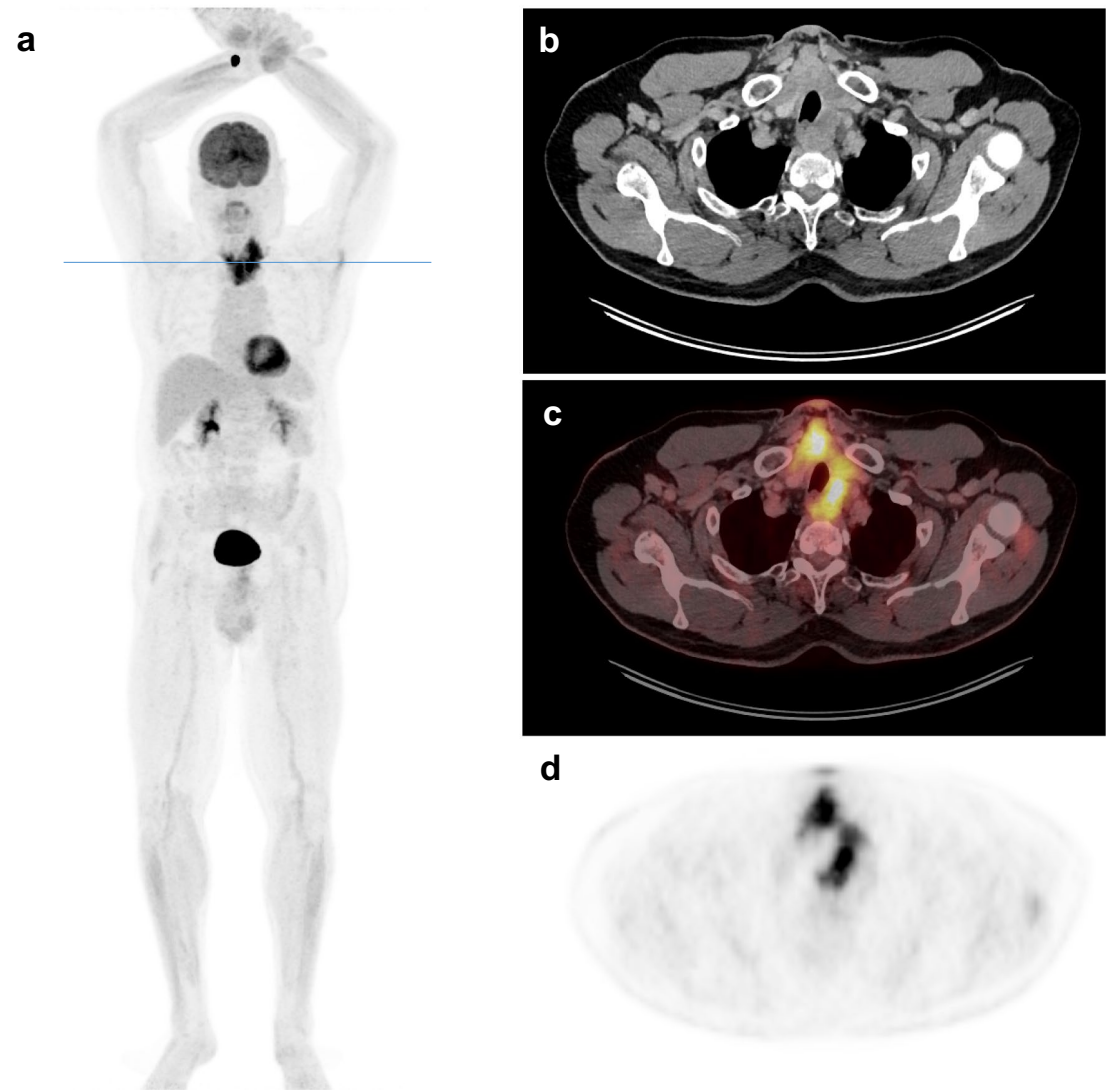
Postoperative FDG-PET/CT scan. Two weeks after surgery FDG-PET/CT scan revealed a 9.6 × 7.1 × 9 cm left cervical tumor mass expanding from the esophagus to the carotid sheath that infiltrates the trachea, the front-edge of the lower cervical vertebrae and the higher thoracic vertebrae. **a** Maximum intensity projections of 18F-FDG-PET. **b** Direct comparison of contrast-enhanced CT, **c** fusion imaging and **d** FDG-PET

Given the diagnosis of highly aggressive MFS and due to its untypical localization, F-18 fluorodeoxyglucose (FDG) positron emission tomography/computed tomography (PET/CT) scan was performed 2 weeks after the operation to find a primary localized elsewhere and to complete tumor staging. Unfortunately, PET/CT-scan excluded a primary somewhere else in the body but demonstrated local recurrence with a 9.6 × 7.1 × 9 cm left cervical tumor mass expanding from the esophagus to the carotid sheath that infiltrated the trachea, the front-edge of the lower cervical vertebrae and the higher thoracic vertebrae (Fig. 3**)**. In addition, a 3-mm measuring nodule in the medial right lung lobe and locoregional lymph node metastases were detected.

**Fig. 4:**
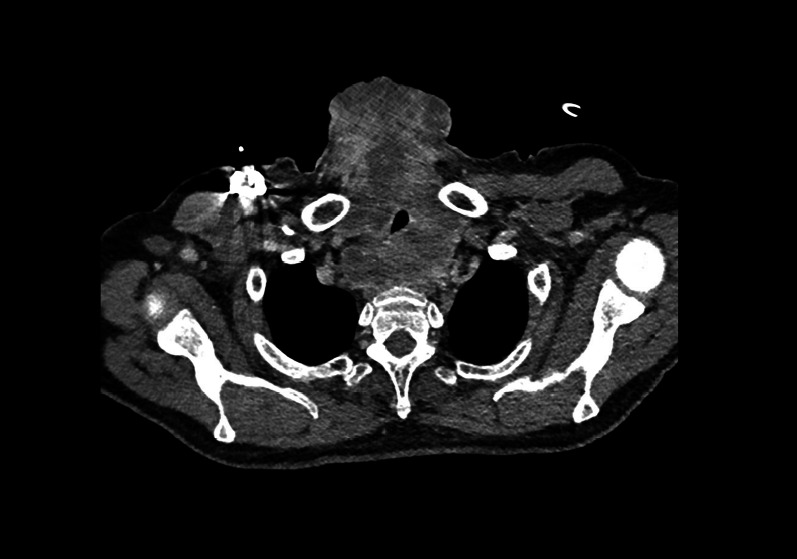
Tumor progression under radiotherapy. Tumor mass in the ventral upper thorax infiltrating the larynx, esophagus and the left common carotid artery

After discussing the case in our multidisciplinary tumor board, an urgent radiotherapy followed by chemotherapy with doxorubicin and ifosfamide was recommended. The patient was admitted to our department of radiation oncology to receive 70 Gy of radiation in fractions of 2 Gy five times a week in a time frame of 7 weeks. Between the radiation sessions, he twice developed dyspnea, supraglottic edema and inspiratory stridor and had to be admitted to the intensive care unit. Both times the symptoms were alleviated with corticosteroid therapy, tracheotomy was avoided, and the patient was able to continue its radiation treatment. Two months after surgery and after the first 30 Gy of radiation, a CT scan of the thorax demonstrated a tumor mass in the ventral upper thorax infiltrating the larynx, esophagus and left common carotid artery (Fig. 4). Unfortunately, due to the tumor's progression, its non-resectability and significantly reduced general condition of the patient, palliative care was initiated.

## Discussion

In this report, we present a rare case of MFS of the thyroid gland. To our knowledge, only 4 cases of primary thyroid MFS have been reported in the literature so far [6–9]. Only one case report described the preoperative use of fine-needle aspiration cytology (FNAC), in which cytology revealed no evidence of MFS but anaplastic thyroid carcinoma [7]. However, considering the rapidly growing tumor with a new onset of hoarseness, we would have chosen our therapeutic strategy even without FNAC, except that we would have performed preoperative imaging to rule out distant metastasis or a primary tumor elsewhere.

MFS represents approximately 5% of all soft tissue sarcomas [3]. The term MFS was first introduced by Angervall et al. in 1977 who described a group of tumors with histiocyte- and/or fibroblast-like cells, nodular and myxoid appearance, plexiform pattern of capillary-like vessels, pleomorphism of the nucleus and a large variation in cellularity, polymorphism and mitotic activity [2]. Until the working group of the WHO's Classification of Tumors of Soft Tissue and Bones found a consensus in 2002, MFS were considered as a part of the group of malignant fibrous histiocytoma (MFH). However, with the introduction of new molecular studies and the progress of immunohistochemistry they became a distinct pathological entity [3]. Based on the degree of cellularity, pleomorphism of the nucleus and mitotic activity, MFS are classified from low- to high-grade differentiated tumors [2]. Several grading systems have been proposed, such as the Brodie and FNCLCC systems, which use four and three grades, respectively, but until today there is no uniformly accepted standard grading system that explicitly applies to MFS [1, 3].

Diagnosis of MFS is based on microscopic characteristics, such as the presence of alternating hypocellular myxoid areas and hypercellular fibrous areas with curvilinear vessels [2, 3]. Although there are no specific immunohistochemical markers for MFS, they may stain positively for vimentin, acid mucins and sometimes SMA or CD34 and are negative for S-100 [2, 10].

Importantly, radiological findings by imaging techniques such as CT scan and magnetic resonance imaging (MRI) may misdiagnose MFS. For example, in CT-scan low-grade MFS may be misinterpreted as a benign tumor and in MRI T2-weight signal MFS may appear as a cystic formation [10]. Moreover, the tail sign, often used for the diagnosis of MFS in MRI, seems to have neither high sensitivity nor high specificity for the differential diagnosis of MFS from other myxoid tumors [11]. Nevertheless, MRI remains currently the imaging method of choice for patients with MFS. Of note, the use of FDG-PET/CT in the diagnosis of MFS is still under debate. Whereas a number of studies demonstrated comparable results between MRI and PET/CT in the identification of locoregional recurrences, its use in the initial diagnosis of a MFS has yet to be determined [12–14].

Irrespective of the grade, MFS shows with up to 61% a high tendency towards locoregional recurrences. Therefore, surgical resection with tumor-free margins, is the standard of care and remains the only chance for cure [15]. Although lymph node metastases are rarely seen at the initial presentation of patients with MFS [16], recent studies demonstrated a frequency up to 31% for lymph node metastases among patients with recurrent distant metastasis [17]. Of note, the study by Sanfilippo et al. [18] reported that 20% of patients who progressed to metastatic disease had previously positive regional lymph nodes. In this respect, the oncologic approach should include prophylactic dissection of the locoregional lymph nodes in addition to resection of the affected thyroid lobe. In our opinion, a total thyroidectomy is not necessary because, in contrast to differentiated thyroid carcinoma, postoperative radioiodine therapy is not indicated.

However, the use of radiotherapy in MFS is still controversial. The existing case series, retrospective studies, and case reports regarding the use of radiotherapy in the treatment of MFS, mostly in an adjuvant setting, show conflicting results [1, 3, 15, 18, 19].

The role of chemotherapy in the treatment of MFS remains unclear [3]. Until today, a randomized clinical trial evaluating the use of chemotherapy specifically in MFS is missing [3]. However, there are a few case series in which chemotherapy is used for the treatment of MFS [5, 18–21].

## Conclusion

The thyroid gland is a very uncommon localization for MFS and only a few cases have been reported in the literature during the past decades. Whereas imaging techniques may be helpful, the gold standard for diagnosis remains histopathology. Radical and wide surgical resection is still the cornerstone in the treatment of MFS and a close clinical follow-up combined with a CT-scan or MRI is mandatory. In addition, adjuvant radiotherapy may play a role in preventing locoregional recurrence. However, the existing data regarding radio- and chemotherapy are from retrospective studies and case series of low evidence. Accordingly, this elucidates the urgent need of multicentric and randomized controlled clinical trials that specifically focus on this aggressive tumor entity.

## Data Availability

All data generated during this case report are included in this article.

## Abbreviations

Anti-Tg: Thyroglobulin-antibody
Anti-TPO: Thyroid-peroxidase-antibody
CT: Computed tomography
FDG: Fluorodeoxyglucose
FNAC: Fine-needle aspiration cytology
FNCLCC: Fédération Nationale des Centres de Lutte Contre Le Cancer
MFH: Malignant fibrous histiocytoma
MFS: Myxofibrosarcoma
MRI: Magnetic resonance imaging
PET/CT: Positron emission tomography/computed tomography
STS: Soft tissue sarcoma
TSH: Thyroid-stimulating hormone
WHO: World Health Organization
